# Supervisor types: which one is your match?

**DOI:** 10.1038/cddiscovery.2016.60

**Published:** 2016-08-01

**Authors:** Tatiana P Soares da Costa

**Affiliations:** 1 Department of Biochemistry and Genetics, La Trobe Institute for Molecular Science, La Trobe University, Melbourne , Victoria, Australia

The relationship with your postgraduate supervisor is one of the most important ones you will have and will be one of the major determinants in the success of your studies.^1^ It is essential that you both communicate well to build a good relationship to become a successful team.^2^

There are a number of different types of supervisors and it is important that you have a good match – just as you may opt for a certain type of partner – otherwise your journey might not be as enjoyable as you want it to be.

## Type 1: The Know-it-all

Telltale signs: you are too familiar with the phrase: ‘I see your point of view but I think you should do the following….’.

Advantages: they probably are a walking encyclopaedia and have a lot of experience in your field.

Disadvantages: their way is not always the only right way or may not be the right way at all.

Compatible with: a student who listens and takes on feedback.

## Type 2: The Absent Supervisor

Telltale signs: always invited to give seminars worldwide, part of a million committees, and even when on campus, the supervisor is never in the office.

Advantages: supervisor probably has a strong profile with an international reputation whose name carries weight when writing a reference. Make sure an absent supervisor is not an unreliable one.

Disadvantages: may not be there when you need them. Also, frequent absence does not necessarily correlate with success – make sure that the absent supervisor really is the one who can give your career a boost by association.

Compatible with: someone who is independent and does not need much guidance. A person who is not afraid to ask questions to other students, postdocs and co-supervisors. Also compatible with someone who takes the initiative to schedule face-to-face or online meetings to discuss the project. At this day and age, you do not have to be physically present in the same place to have meetings.

## Type 3: The Perfectionist

Telltale signs: someone who criticises every aspect of your work. A supervisor who, although using track changes to provide you feedback, turns your document more red than black. Usually someone who is hard to impress.

Advantages: will be able to help you achieve the best results possible.

Disadvantages: can be overly critical and destroy confidence.

Compatible with: students who are also perfectionists. Students who would like a large amount of guidance before developing their own independence.

## Type 4: The Very Hands-on Supervisor

Telltale signs: you get calls/messages after hours from your supervisor. Your supervisor writes you e-mails late at night that need to be answered within 12 h. You feel like they are always breathing down your neck.

Advantages: they tend to be very involved with every aspect of your project, which may be useful when you encounter any problems.

Disadvantages: may stifle independence. Remember, postgraduate studies are an evolving process towards independence as a researcher, so by the end, you need to be in control.

Compatible with: someone who likes to have constant guidance. Also suitable with a student who can set boundaries and is not afraid to let the supervisor know of their working hours. Even though postgraduate studies are not a 9-to-5 job, you should still be entitled to some time off. Remind your supervisor that you have a life outside of work.

## Type 5: The Pessimist

Telltale signs: supervisor starts every meeting with ‘This will probably not work but you should try it anyway’.

Advantages: at least they are not going to be disappointed if something does not go according to plan. They certainly will not fill you with false hope!

Disadvantages: can really erode your confidence and enthusiasm, particularly if things are not going well in the first place.

Compatible with: an optimist.

## Type 6: The Friend

Telltale signs: someone who will tell you all the things you want to hear.

Advantages: supervisor will understand life events, will impact on your work, and will be very sympathetic and talk through practical solutions.

Disadvantages: may sometimes have difficulty delivering bad news or much needed criticism.

Compatible with: students who need emotional support.

## Type 7: The Coach

Telltale signs: supervisor is involved in the growth of the student in all aspects of their life. Supervisor will encourage you to go on training courses, to present your work and get you involved in community/departmental engagements.

Advantages: you will be involved in different aspects of academia that will help expand your resume. You will also acquire other transferable skills that will be useful in nonacademic fields.

Disadvantages: do not forget that your research should still be your main focus.

Compatible with: someone who wants to have an all-round experience during their studies. They want to be doing cutting edge research in the lab, but also be involved in other activities to expand their skill set. A multitasker would be preferable.

**Figure 1:**
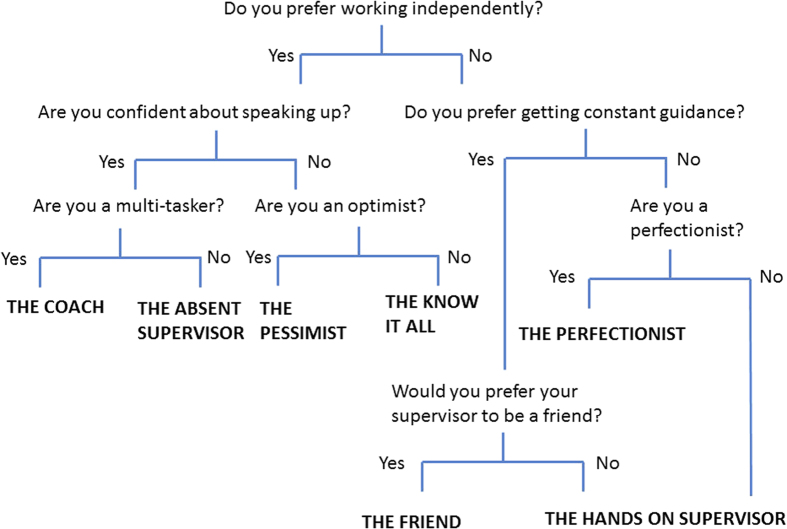
If you cannot figure out what your perfect supervisor match is, hopefully this quiz can!

There you have it. Which type is your perfect match? If you are still unsure, try the quiz above as a guide to find your match (Figure 1). It is important to find someone who is compatible because they are not only going to be your supervisor for a few years while you are studying; they may also be your mentor for the rest of your life. Importantly, they will probably be the first person to be called when you are applying for your dream job after you graduate. It is critical to establish good working practices early and manage your expectations.^3^ Make sure you are both on the same page about your milestones and direction of your project, and make necessary changes to the working relationship if needed.^4^ Bear in mind that your needs and expectations may vary depending on the stage of your studies.^5^ But if you have managed to land yourself a supervisor that is not your match, all is not lost. Most students have more than one supervisor these days for a reason, utilise them! You can also get useful guidance and mentorship from other people in your circle. There may be a co-supervisor, a postdoc or a more senior lab member that you can draw upon for advice/guidance. And remember that sometimes opposites attract!
